# Exploring Label-Free Imaging Techniques with Copper Sulfide Microspheres for Observing Breast Cancer Cells

**DOI:** 10.1021/acsomega.4c04154

**Published:** 2024-08-28

**Authors:** Lutvi Vitria Kadarwati, I-Hsin Lin, Yu-Shan Huang, Yu-Yang Lee, Shin-Cyuan Chen, Chia-Lin Chung, I-Jan Chen, Jia-Yeh Wang, Sibidou Yougbaré, Tsai-Mu Cheng, Tsung-Rong Kuo

**Affiliations:** † Graduate Institute of Biomedical Optomechatronics, College of Biomedical Engineering, Taipei Medical University, Taipei 11031, Taiwan; ‡ School of Biomedical Engineering, College of Biomedical Engineering, Taipei Medical University, Taipei 11031, Taiwan; § Graduate Institute of Nanomedicine and Medical Engineering, College of Biomedical Engineering, Taipei Medical University, Taipei 11031, Taiwan; ∥ Southport Corporation, New Taipei City 22175, Taiwan; ⊥ Institut de Recherche en Sciences de La Santé/Direction Régionale du Centre Ouest (IRSS/DRCO), Nanoro BP 218, 11, Burkina Faso; # Graduate Institute for Translational Medicine, College of Medical Science and Technology, Taipei Medical University, Taipei 11031, Taiwan; ¶ Taipei Heart Institute, Taipei Medical University, Taipei 11031, Taiwan; ∇ Cardiovascular Research Center, Taipei Medical University Hospital, Taipei Medical University, Taipei 11031, Taiwan; ○ International Ph.D. Program in Biomedical Engineering, College of Biomedical Engineering, Taipei Medical University, Taipei 11031, Taiwan; ⧫ Stanford Byers Center for Biodesign, Stanford University, Stanford, California 94305, United States

## Abstract

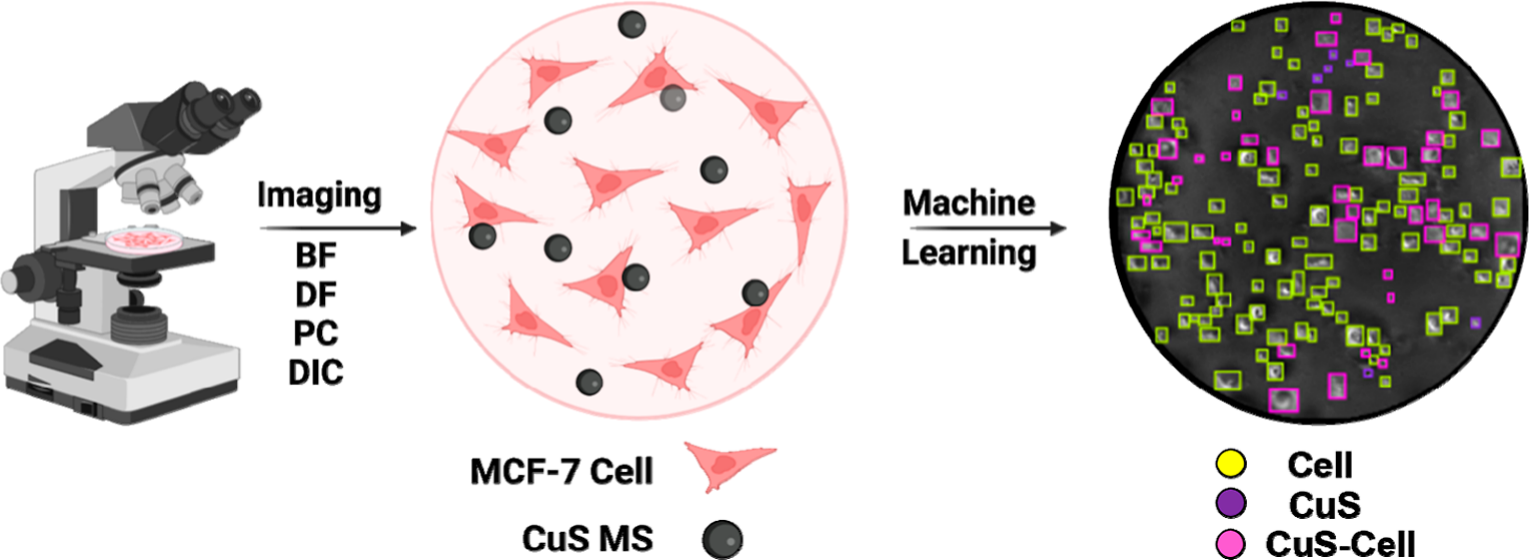

A single breast cancer is a prevalent form of cancer, affecting over 2.3 million women worldwide, as reported by the World Health Organization. Recently, researchers have extensively explored the utilization of biomaterials in breast cancer theranostics. One notable biomaterial being investigated is various structures of copper sulfide (CuS). In this work, a microsphere (MS) structure composed of CuS was employed for label-free imaging of MCF-7 breast cancer cells and normal Vero cells, respectively. Various label-free imaging techniques, such as bright field, dark field, phase contrast (PC), and differential interference contrast (DIC), were employed to capture images of CuS MSs, cell, and intact CuS MSs within a cell. The study compared the outcomes of each imaging technique and determined that DIC imaging provided the highest resolution for cells incubated with CuS MSs. Furthermore, the combination of PC and DIC techniques proved to be effective for imaging breast cancer cells in conjunction with CuS MSs. This research underscores the potential of CuS MSs for label-free cell detection and emphasizes the significance of selecting appropriate imaging techniques to attain high-quality images in the field of cell observation.

## 1 Introduction

Based on data from the World Health Organization, breast cancer is a highly prevalent cancer that affects more than 2.3 million women in the world, due to certain genetic and environmental factors among others.^1^ Metastasis has become the most important factor for breast cancer-related deaths due to the lack of effective drugs for treating such cases.^2−5^ Thus, identifying prognostic markers is essential for predicting metastasis and therapeutic targets.^6−9^ The structure and morphology of cells can be analyzed through optical microscopy, a crucial step in studying cell behaviors and their interactions with the microenvironment and subcellular organelles.^10−15^ Numerous microscopy systems are employed to identify and evaluate bacteria, cells, and combinations of in vitro models and materials. With a binocular microscope, researchers have examined the structure and health of oocytes for oocyte assays and developed algorithms for stereoscopy to study the 3D deformation and movement of amoeboid cells.^16,17^ Optical projection tomography is a highly efficient technique for studying cells and tissues in three dimensions, allowing both quantitative and qualitative analyses of hydrogel cell cultures and enabling the examination of cell responses in various hydrogel formulations through 3D imaging.^18^ Additionally, the morphology and adhesion images of cell membranes in neutrophils have been analyzed using confocal laser scanning microscopy combined with AFM optics to investigate the duration of reactive oxygen species during cell activation.^19^

Regarding contrast enhancement techniques for optical microscopy, bright field (BF), dark field (DF), phase contrast (PC), and differential interference contrast (DIC) techniques have evolved to enhance the contrast in optical microscopy to identify cells and their modulation due to the presence of biomaterial agents, which are typically disabled for detection in visible light without labeling or staining.^20−24^ Initially, BF optical microscopy was the first bioimaging technique to evaluate the specimen morphology in two-dimensional samples. BF images can provide 300× images more rapidly than human labeling and obviously present intracellular regions and cell boundaries.^25−29^ In addition, it has the ability to record cell photographs with less advanced preparation and can capture dynamic cell activities during a period of time.^30−32^ Besides being an inexpensive microscopic method, it produces less phototoxicity and photodamage than fluorescence microscopy.^33^ However, bright-field images show low contrast in terms of phase objects between the cell and background, which affects difficulties with the segmentation process.^34^

DF optical microscopy is a method that uses a dark background to easily detect and legibly show light-scattering properties of objects due to the contrast.^35,36^ It is known as a great option in addition to BF microscopy to investigate cells without a lightening process and is suitable for discovering single molecules.^37−39^ Using DF microscopy, bacterial activity in complex samples can specifically be investigated, and the number of immobilized bacteria can be counted.^40^ Although it is commonly used to observe chemical reactions and investigate the distribution of nanoparticles within biomaterials, DF microscopy is inappropriate for detecting embedded metal nanoparticles in cells. Additionally, sample preparation on thin slides is necessary, which can block the direct imaging of cultured cells, and it is tough to discard unwanted parts of nanoparticles, such as the bubbles, which potentially appear during the dilution process.^41,42^

PC microscopy is an enhancement technique that refers to the principle of transparent objects without a defocusing stage of the image.^43^ It has no phototoxicity effect, and thus samples are safe, and long-term imaging is allowed for further applications.^44−47^ Defocused images of cell cultures can be overcome by emulating contrast changes so that figures can be segmented. Moreover, it not only provides the ability to demonstrate the imaging of biological cells, polymeric nanostructures, and transparent meta-surfaces, but PC microscopy also is applicable to analog optical computing and image processing for further information.^48^ It perfectly fits label-free imaging, which is defined as the visualization of cell dynamics, including live mobile organisms.^49^

DIC microscopy is another technical option that possesses the possibility to visualize invisible structures and organelles of cells in a three-dimensional appearance, which are undetectable with BF microscopy.^50−54^ DIC microscopy shows the gradient of the optical path at both high and low spatial frequencies through a sample.^55^ It provides high-resolution imaging for unstained, transparent, live organisms by optimizing the contrast and inherent optical sectioning.^56^ The ultrastructure of microbial and epithelial surfaces can be visualized with this type of microscopy without fluorescence.^57^ It is convenient to use DIC microscopy as fluorescent dyes or labeled markers are unnecessary.

Moreover, some research has deployed contrast enhancement techniques to obtain a suitable image of each experiment, particularly by observing nanoparticles with living cells. Meanwhile, most studies prior to this merely used single or double methods independently to compare the results. In this case, in addition to obtaining individual images from each technique, we also combined the two techniques into one picture to generate images with varying quality and processing with machine learning, which have not been previously reported.

In this study, we observed the interaction of CuS MS intake with breast cancer cells by using optical technology and investigated the best technique for displaying high-resolution images from a microscope. We synthesized copper sulfide microspheres (CuS MSs) and examined them in in vitro models, specifically a breast cancer cell line (MCF-7) and normal cell line (Vero cells), to study cell viability. In terms of optical properties, CuS possesses a high indication as a contrast agent in photoacoustic imaging (PAI) of biological tissue or organs, act as a multipurpose theranostic agent, and actively seeks to eradicate breast cancer.^58−60^ Additionally, the high absorption capacity of CuS MSs indicates that it is an excellent candidate for photoacoustic tomography (PAT) and acts as a visible light harvester.^61,62^ It can be compared with a gold nanostructure, which is difficult to prepare in small size and seek optical imaging.^63^ The microparticle of CuS was specifically chosen for analysis because it was suitable for monitoring interactions between cells and particles by using an optical microscope. Typically, the particle size was observed to range from 0.5 to 100 μm, while the average animal cell has a diameter ranging from 10 to 20 μm.^64^ Furthermore, we visualized cell images using four different types of label-free imaging techniques, including BF, DF, PC, and DIC, and their combinations, before undertaking image analysis. In the last stage, the performance of each imaging technology was conducted using an object detection method.

## 2 Materials and Methods

### 2.1 Materials

Copper(II) nitrate trihydrate [Cu(NO_3_)_2_·3H_2_O, 99%, for analysis], thiourea (CH_4_N_2_S, 99+%, for analysis), and ammonium hydroxide (28–30 wt % solution of NH_3_ in water) were supplied by Acros Organics (Geel, Belgium). Nitric acid (HNO_3_, ACS reagent, 70%), ethanol (C_2_H_5_OH), and paraformaldehyde (PFA) were purchased from Sigma-Aldrich (St. Louis, MO, USA). All chemicals were used without further purification.

### 2.2 Synthesis of CuS MSs

Deionized water (40 mL) was added to 1 mmol of Cu(NO_3_)_2_·3H_2_O in a test tube and stirred for 30 min on a hot plate stirrer. Then, 3 mmol of thiourea was added to the solution, and the mixture was continuously and vigorously stirred for 15 min until color differences were visible, in particular, bluish-green to pale-white. The mixture was shifted to an autoclave reactor and sealed in a hot-air oven for 24 h, and the temperature was maintained at 150 °C in order to obtain a homogeneous solution. In the following stages, the autoclave reactor was cooled to room temperature, and the black precipitate that appeared was washed off several times with deionized water and ethanol. The final product was dried at 60 °C overnight and was sonicated for further application.

### 2.3 Cell Culture

The MCF-7 breast cancer cell line (ATCC HTB-22) and human normal kidney Vero cell line (ATCC CCL-81) are commonly used for cell culture. Cells were seeded at 8 × 10^3^ cells/well in a 96-well plate and cultured in fresh Dulbecco’s modified Eagle medium (DMEM) supplemented 10% fetal bovine serum (FBS) and 1% antibiotic. All cells were maintained in an incubator with 5% CO_2_ and at 37 °C for 24 h.

### 2.4 Cell Viability Assay

This research used a crystal violet assay to examine the viability of MCF-7 and Vero cells as it is an efficient method for in vitro cytotoxicity studies. On the first day, we prepared and seeded cells into a 96-well plate with 8 × 10^3^ cells/well and added 10 μL of DMEM. Before storing in an incubator for 24 h, we checked the morphology of cells with a microscope. Cells were starved on the second day. Cells were treated with the compound on the third day. Initially, we prepared HNO_3_ as the solvent for CuS MSs and put 100 μL of solution into 900 μL of DMEM. We took 100 μL of the solution from the first dilution and added it to new medium six different times and took the last four solutions as different concentrations. After that, 100 μL of the solution was added to each well, shaken on a shaker for 10 s, and then placed in an incubator for 48 h. In the following stage, a 96-well plate was washed twice with phosphate-buffered saline. PFA was used to fix cells on the plate, and it was allowed to sit for 1 h at room temperature. It was removed and stained with 50 μL of crystal violet dye in each well for 30 min. The dye was removed, and cells were washed and dried before checking the value of the optical density at 570 nm with an enzyme-linked immunosorbent assay (ELISA) reader.

### 2.5 Cell Imaging

MCF-7 cells were seeded in a single cell culture plate and cultured in fresh DMEM containing 10% FBS and 1% antibiotic in an incubator at 37 °C with 5% CO_2_. After incubating for 24 h, the cells were treated with 100 μg/mL CuS solution, which was diluted by doubled-deionized water, and a whole plate was observed on the next day by using a FOV 900 μm Fourier plane modulated microscope (JadeDot-FM, Southport Co., New Taipei City, Taiwan), which can perform digital and instant switching between BF, DF, PC, DIC, and hybrid image modes without any phase plate operation and the need to transfer the sample from one platform to another.

### 2.6 Image Processing

In the preprocessing stage, raw images were processed using MATLAB in order to obtain histogram distribution and the image texture by calculating the entropy. Moreover, cell detection was observed by Roboflow, an online software for computer vision models to annotate the cells.

## 3 Results and Discussion

### 3.1 Morphological Characterizations of CuS MSs

**Figure 1:**
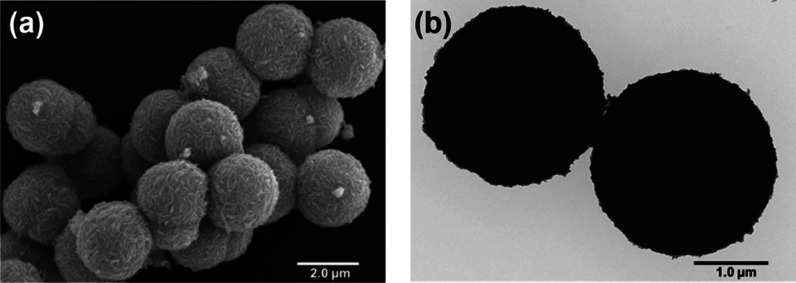
(a) SEM image and (b) TEM image of copper sulfide microspheres (CuS MSs).

The morphology of the synthesized CuS MSs was examined by scanning electron microscopy (SEM) and transmission electron microscopy (TEM). The former was used to provide the 3D structure of the outer surface, while a two-dimensional projection of the inner surface of the sample was provided by the latter.^65−68^ In the SEM image shown in Figure 1a, the CuS MSs appeared spherical with an average size of approximately 2.42 μm. As shown in the TEM image in Figure 1b, the CuS MSs exhibited exceptional uniformity in their spherical shapes, corroborating the findings from the SEM image. Furthermore, the diameter of the spheres was confirmed to range between 2.38 and 2.47 μm. The microscale size of the CuS MSs was chosen because it is within the observable range for a microscope. Moreover, the composition of CuS MSs was measured by an energy-dispersive X-ray (EDX) spectroscopic analysis, which revealed that CuS consisted of copper (Cu, 68.21 wt %) and sulfur (S, 31.79 wt %). For CuS MS, the atomic ratio of Cu to S was calculated to be ∼1. The EDX elemental mapping of CuS MSs is provided as Figure S1 in the Supporting Information. Overall, the morphological characterizations demonstrated the successful preparation of CuS MSs with spherical shape, average size of ∼2.48 μm, and homogeneous distribution of Cu and S elements.

### 3.2 Optical and Structural Characterizations of CuS MSs

**Figure 2:**
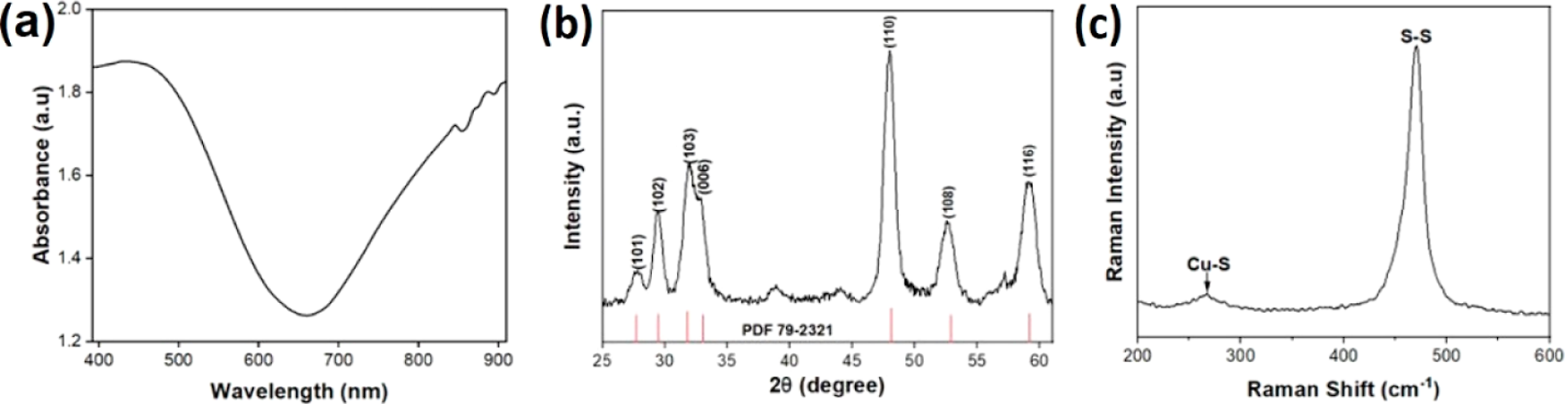
(a) UV–vis–NIR spectra of CuS MSs. (b) XRD spectra of CuS MSs. (c) Raman spectra of CuS MSs.

Ultraviolet/visible/near-infrared (UV–vis–NIR) spectroscopy was utilized to examine the absorption of CuS MSs. As shown in Figure 2a, the CuS MSs showed strong absorption from 600 to 800 nm, corresponding to the characteristic absorption of CuS, which revealed the photocatalytic activities and photothermal capabilities.^69^ Furthermore, X-ray diffraction (XRD) analysis was used to determine the crystallographic structure and validate the formation of CuS MSs. In the XRD spectrum shown in Figure 2b, the positions of reflection peaks were observed at 27.79, 29.37, 31.96, 32.82, 47.92, 52.56, and 59.09°, which were associated with Miller indices at (1 0 1), (1 0 2), (1 0 3), (0 0 6), (1 1 0), (1 0 8), and (1 1 6), respectively.^70^ The XRD spectrum of CuS MSs indicated that CuS MSs had a crystalline structure and a hexagonal arrangement of Cu and S atoms.^71^ Moreover, Raman spectroscopy was used to differentiate chemical properties, such as the structure and molecular interactions, in the CuS MSs. As shown in the Raman spectrum in Figure 2c, CuS MSs presented the lowest peak at 269 cm^–1^ followed by 471 cm^–1^ as a pointed peak to validate the formation of CuS. Both peaks indicated that CuS MSs had a stretching vibration mode of a hexagonal structure on the Cu–S and S–S lattice.

**Figure 3:**
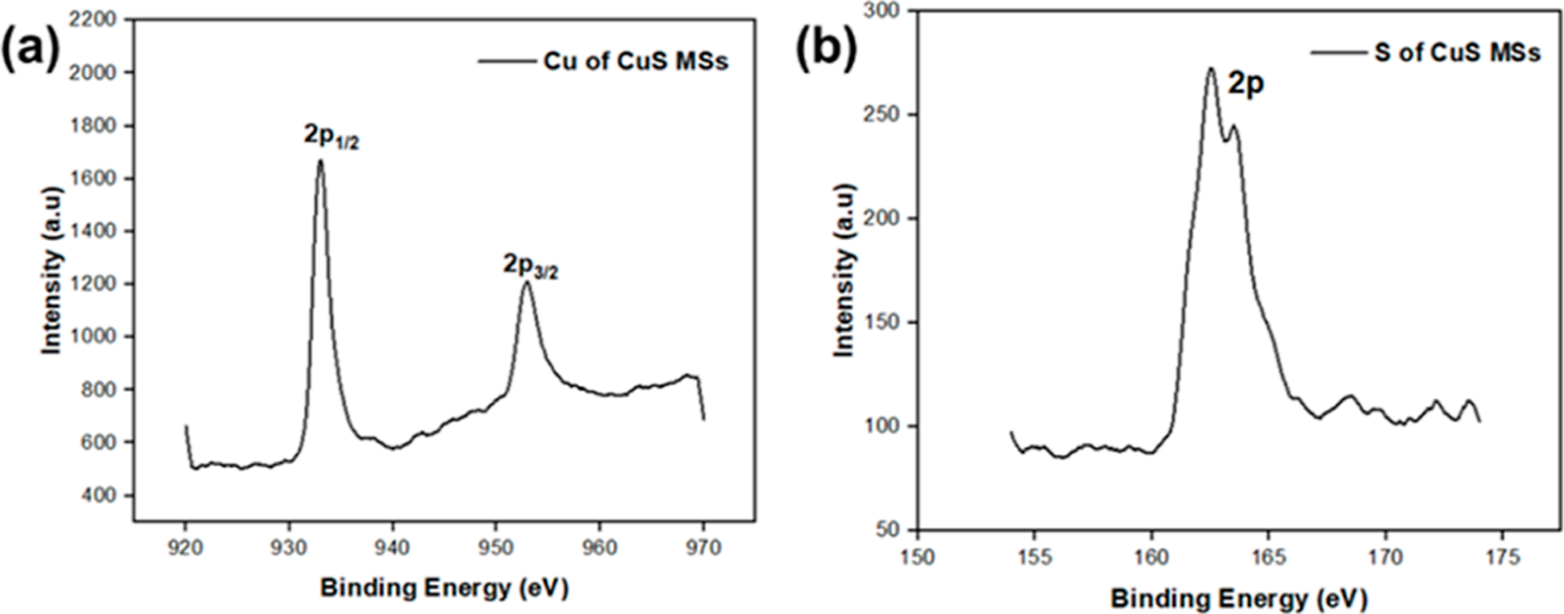
XPS spectra of CuS MSs. (a) Cu 2p MSs and (b) S 2p MSs.

X-ray photoelectron spectroscopy (XPS) was conducted to further investigate the properties of the CuS MSs. The distinctive XPS spectra of each element were determined by their specific energy levels and associated binding energy transitions. In Figure 3a, the Cu spectrum of CuS MSs exhibited binding energies at 932.8 and 952.9 eV, corresponding to the Cu 2p_1/2_ and Cu 2p_3/2_ states, respectively.^69^ The Cu 2p_3/2_ spectrum of CuS (covellite) displays a single peak at 932.4 eV, characteristic of the sole oxidation state of Cu(I).^72^ Additionally, the S spectrum of CuS MSs showed two spin–orbit peaks significantly different in binding energy positions: 162.2 eV for the S 2p_1/2_ state and 163.3 eV for the S 2p_3/2_ state, indicating the presence of S^2–^ and S_2_^2–^. The XPS analysis of CuS MSs was consistent with and corroborated by the XRD and Raman data obtained in this study. These findings are further supported by the existing literature.

### 3.3 Cytotoxicity Study of CuS MSs

**Figure 4:**
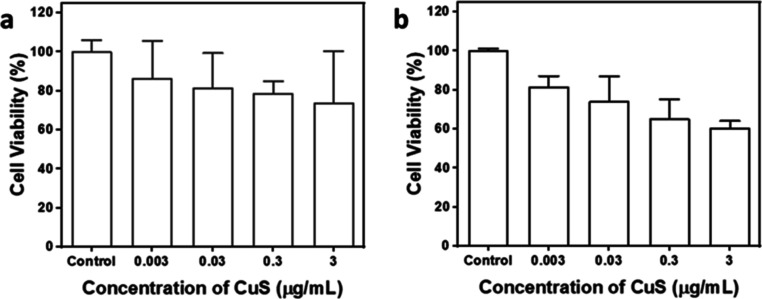
Biocompatibility studies of CuS MSs at various concentrations in the MCF-7 and Vero cell lines. (a) Viability test using the MCF-7 breast cancer cell line and (b) normal Vero cells from a monkey kidney.

Before being introduced in clinical use, the biocompatibility of CuS MSs was examined using a crystal violet assay to quantify cell death and determine cell proliferation after being stimulated with a death-inducing agent.^73^ As shown in Figure 4a, the CuS MSs revealed excellent cell viability after treatment with the MCF-7 cell line, with above 80% for the concentrations from 0.003 to 3 μg/mL. For Vero cells, the cell viabilities of CuS MSs were slightly decreased for the concentrations ranging from 0.003 to 3 μg/mL, as shown in Figure 4b. As observed from the cell cytotoxicity study, CuS in microsphere shape exhibited no significant impact of cytotoxicity for MCF-7 cells. Based on the results of cell cytotoxicity, CuS MSs had good biocompatibility, and nontarget cells would not be influenced by the CuS MSs.^74^ Hence, the MCF-7 cell line was chosen to capture the optical image with CuS MSs attachment.

### 3.4 Image Processing and Analysis

**Figure 5:**
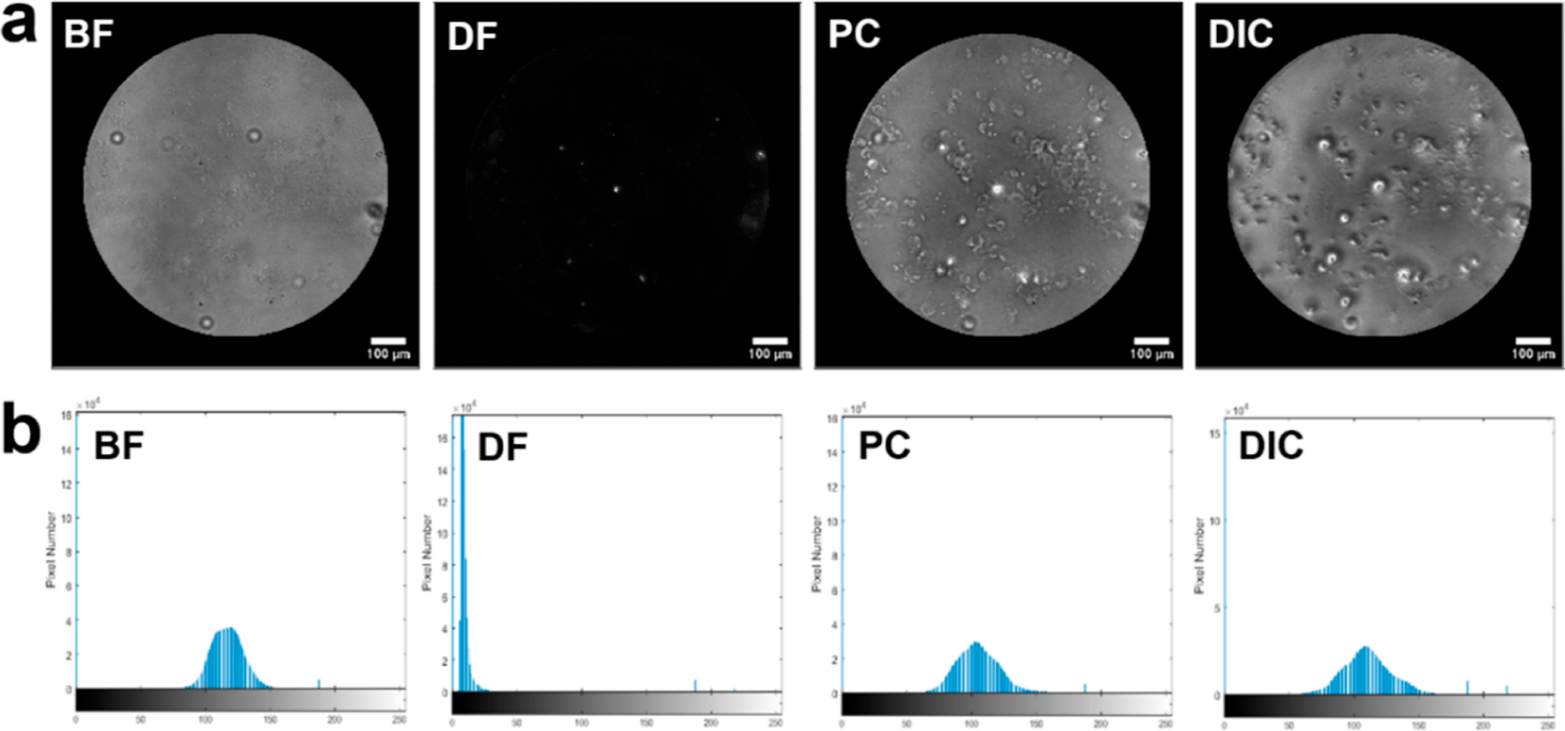
(a) Raw images of BF, DF, PC, and DIC. (b) Histograms show grayscale distributions for BF, DF, PC, and DIC. In each histogram, the vertical color panes indicate the number of detected pixels in the range of 0–255. Abbreviations: BF: bright field; DF: dark field; PC; phase contrast; DIC: differential interference contrast.

We observed proposed images analyzing MCF-7 breast cancer cells with different contrast enhancements. The physical and chemical features of the CuS MSs were determined by the particle distribution in the images.^75^ The original images were grayscale images, and the size of each image was 861 × 863 pixels with a 96-pixel density (Figure 5a). The image contrast information was computed by using MATLAB, and a histogram was displayed as a step to quantify the pixel numbers of the images. A contrast histogram has a range of gray-level values from 0 to 255 and represents divergence of the target and background. Figure 4b shows that the DIC image had a large grayscale range from 50 to 170 followed by PC, and a comparison of BF and DF images indicated that it had high contrast. The grayscale and the contrast between target and background were enhanced.

Moreover, the texture of the image could feasibly and easily be detected by human vision, while this matter is a complicated stage in image processing to be described by the machine vision domain. The texture in image processing is defined as a local spatial variation of the brightness intensity of pixels.^76^ That refers to variations in intensity values, such as the grayscale level, which clarify the shade of gray in each pixel of image. It is normally from black to white, 0–255, respectively. Statistically, characterization of a texture image can be obtained by measuring the entropy as a texture analysis and is exerted to harmonize high-resolution images.^77^ Entropy is a randomness metric that is important in describing texture related to intensity distribution.^78^ The average information per source output relies on the random events of source from a discrete set of probable events with associated probabilities which according to the entropy equation below.
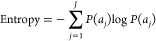


The quantity of information and identification of zones in the image can be assessed by measuring entropy for image segmentation. Converting grayscale or color image into a binary image, called by threshold, corresponds to the maximum entropy of images. The conversion of the original image into grayscale is required before identifying the entropy.

**Figure 6:**
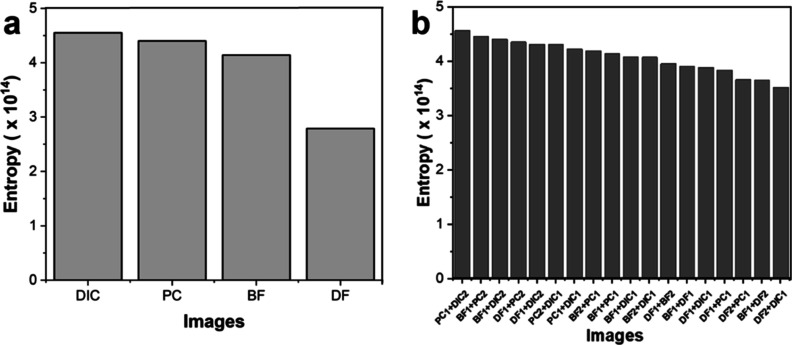
(a) Entropy of four basic enhancement techniques, including DIC, PC, BF, and DF. (b) Entropy of combined two contrast techniques.

In terms of entropy (Figure 6a), DIC and PC images of Figure 4a showed the highest values of entropies at 4.55 × 10^14^ and 4.40 × 10^14^, respectively. Together, these outcomes show excellent image quality and yield a positive result. Both of the entropy values DIC and PC were comparable with BF and DF at 4.14 × 10^14^ and 2.79 × 10^14^, successively, where BF was the lowest entropy value and indicated minimum resolution image. In order to obtain an elevated quality of the images, combinations of basic contrast techniques were further used to evaluate their entropies. In Figure S2, the images combined two basic contrast techniques, including DIC + PC, DIC + BF, DIC + DF, PC + BF, PC + DF, and BF + DF were calculated. As shown in Figure 6b, PC integrated with DIC offered an obvious texture that displayed cells and materials through the image with an entropy of 4.57 × 10^14^.

### 3.5 Visualization Analysis

**Figure 7:**
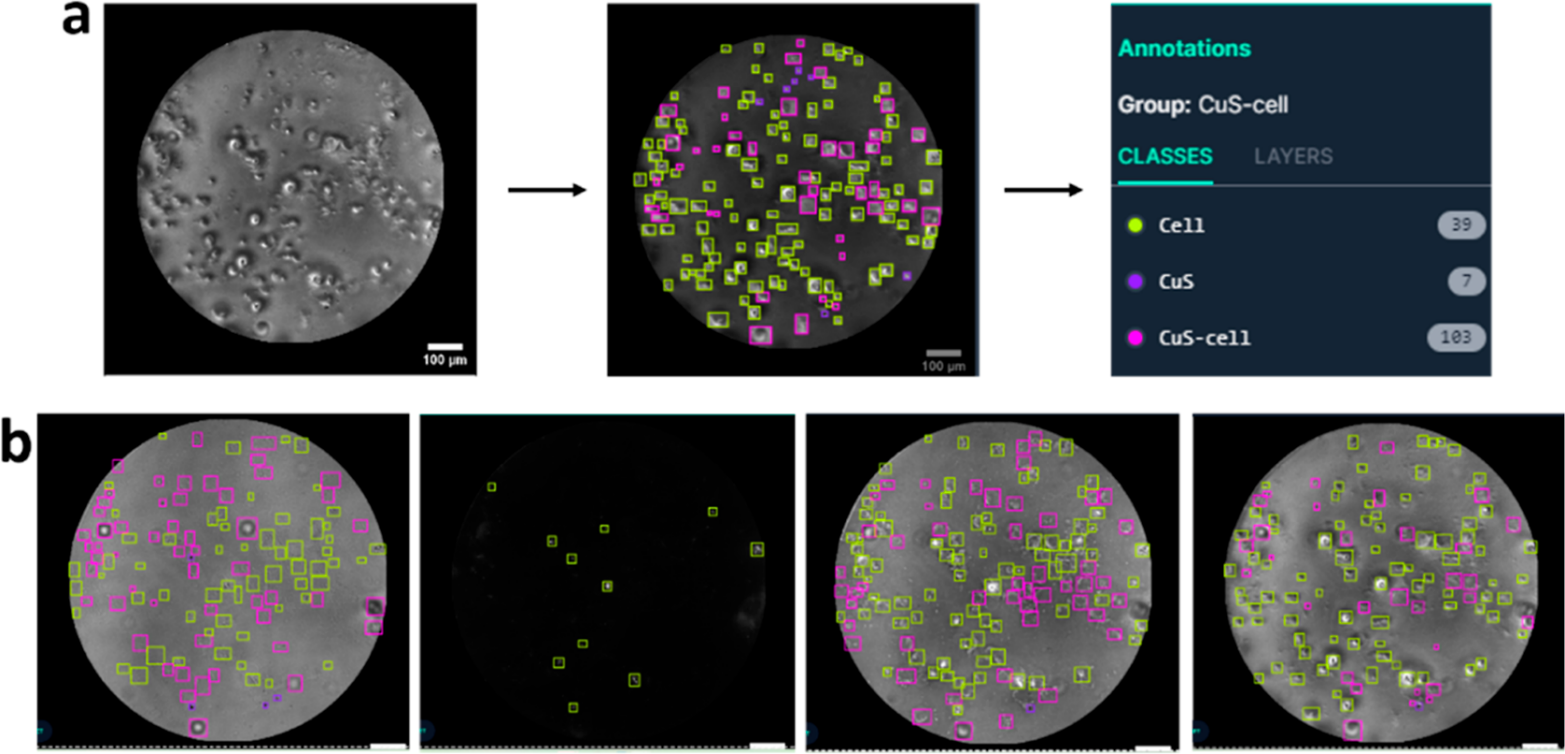
Image processing scheme. (a) Annotation process of a raw image. (b) Image labeling appearance. The scale bar is 100 μm.

Visual analysis is an essential stage for recognizing each element of a cell image and can be analogous to cell detection. Based on this step, it was easy for researchers to distinguish different cells with the contrast technique. Herein, we classified three groups, including CuS MSs, cells, and intact CuS MSs within a cell. In order to calculate amounts of three groups in each image, annotation was manually applied to the images using Roboflow as a labeling online software and categorized into three different colors, signifying three classes (Figure 7a). Image annotation is defined as a labeling process to recognize, count, or track an object in computer vision. After annotating the image, results showed the number of counted elements which depicted the quality of the texture image. The appearance of each image after annotating can be seen in Figure 7b, and the DF image could solely visually recognize a small number of objects of the CuS MS within a cell.

**Figure 8:**
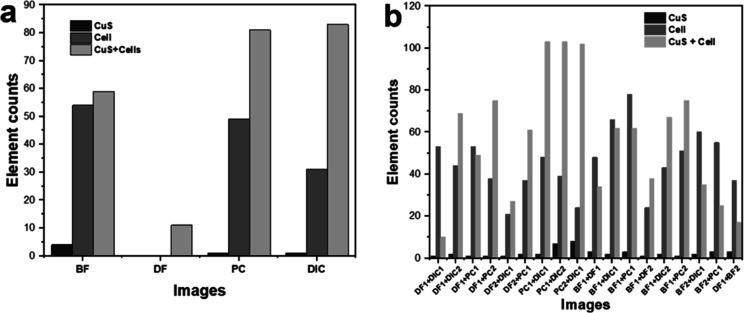
(a) Element counts of four basic techniques. (b) Results of particle counts detected from combinations of different contrast enhancement techniques.

Accordingly, this study aimed to compare the number of cells from multiple contrast microscopic images and verified that DIC and PC images showed high numbers of cell and CuS MS observations with 83 and 81 objects (by Roboflow), respectively (Figure 8a). However, CuS MSs were invisible in DF images as they were of a similar color as the background. On the other hand, the combination of DIC and PC revealed significant results by revealing 103 objects, which indicated that those techniques were suitable for displaying quality images as shown in Figure 8b. This can be follow-up information to prove better techniques for enhancing contrast images. After annotating and counting the detected cells, we trained the image by deploying TensorFlow in Roboflow, and these showed desirable performances. Training a model is required to recognize each object and classify it correctly. After deploying the initial 22 raw images, we processed a total of 584 images by applying autoorientation and tiling techniques during the preprocessing stage, which are commonly used to improve accuracy when handling small objects. The data set was divided into 540 images for training, 24 images for validation, and 20 images for testing. To evaluate the model, we assessed its performance using metrics such as precision, recall, and mean average precision (mAP). Most objects in the image could be distinguished and accurately classified into different groups, achieving a precision of 90.3% and a mAP of 84.5%. These metrics are commonly used to assess and quantify the performance of computer vision models, with higher mAP values indicating greater accuracy.^79^ Such criteria are typically employed to evaluate the effectiveness of the computer vision models.

## 4 Conclusions

In conclusion, the structural and optical characterizations demonstrated the successful preparation of CuS MSs via a hydrothermal approach. The CuS MSs were confirmed with high biocompatibility for breast cancer cell line MCF-7 and normal cell line Vero based on the crystal violet assay. The CuS MS was further utilized as the contrast agent for cell imaging, specifically for cancer cells. The enhancement of microscopic techniques was established to achieve the exceptional image quality of cells and agents. Here, we propose a promising option for a technique of live cell imaging based on four commonly used methods, namely, BF, DF, PC, and DIC. Experimental results showed that DIC and PC were proper approaches to precisely obtain cell segmentation followed by a combination of these two techniques. Numbers of cells and materials attached to cells can easily be annotated, and these methods provide good aspects in terms of contrast and texture. This research can guide the development of microscopes by integrating DIC and PC approaches to obtain high-quality images. This work also focuses on utilizing machine learning to assess the quality of captured images by using object detection methods with deep learning features. Automating the annotation process will enable users to efficiently train a large number of samples simultaneously, saving time.

## Data Availability

All data are available throughout the manuscript and supporting files.

## Supporting Information Available

EDX elemental mapping of CuS Ms and images combining two basic contrast techniques, including DIC + PC, DIC + BF, DIC + DF, PC + BF, PC + DF, and BF + DF (PDF)
