# Supplementary material for: Exploring Label-Free Imaging Techniques with Copper Sulfide Microspheres for Observing Breast Cancer Cells
Source: ACS Omega. 2024 Aug 28;9(36):37882–90. doi: 10.1021/acsomega.4c04154 (PMC11391449; doi:10.1021/acsomega.4c04154)
Supplement: Supplementary file 1 — ao4c04154_si_001.pdf [file ao4c04154_si_001.pdf]

Supporting Information

Exploring Label-Free Imaging Techniques with Copper Sulfide Microspheres for Observing Breast Cancer Cells

Lutvi Vitria Kadarwati[1,a], I-Hsin Lin[2,a], Yu-Shan Huang[3,a], Yu-Yang Lee[4], Shin-Cyuan Chen[4], Chia-Lin Chung[4], I-Jan Chen[4], Jia-Yeh Wang[4], Sibidou Yougbaré[5], Tsai-Mu Cheng[6,7,8,*] and Tsung-Rong Kuo [3,9,10,*]

[1] Graduate Institute of Biomedical Optomechatronics, College of Biomedical Engineering, Taipei Medical University, Taipei 11031, Taiwan

[2] School of Biomedical Engineering, College of Biomedical Engineering, Taipei Medical University, Taipei 11031, Taiwan

[3] Graduate Institute of Nanomedicine and Medical Engineering, College of Biomedical Engineering, Taipei Medical University, Taipei 11031, Taiwan

[4.] Southport Corporation, New Taipei City 22175, Taiwan;

[5] Institut de Recherche en Sciences de La Santé/Direction Régionale du Centre Ouest (IRSS/DRCO), Nanoro BP 218, 11, Burkina Faso; ysibidou@gmail.com

[6] Graduate Institute for Translational Medicine, College of Medical Science and Technology, Taipei Medical University, Taipei 11031, Taiwan

[7] Taipei Heart Institute, Taipei Medical University, Taipei 11031, Taiwan

[8] Cardiovascular Research Center, Taipei Medical University Hospital, Taipei Medical University, Taipei 11031, Taiwan

[9] International Ph.D. Program in Biomedical Engineering, College of Biomedical Engineering, Taipei Medical University, Taipei 11031, Taiwan

[10] Stanford Byers Center for Biodesign, Stanford University, Stanford, CA 94305, USA

* Correspondence: tmcheng@tmu.edu.tw (T.-M.C.); trkuo@tmu.edu.tw (T.-R.K.)

[a] These authors contributed equally to this work

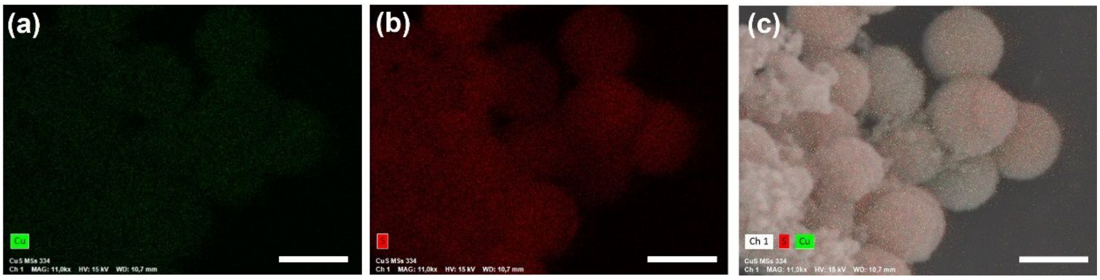

Figure S1. EDX elemental mapping of CuS Ms (a) Cu mapping, (b) S mapping, and (c) overlay map.

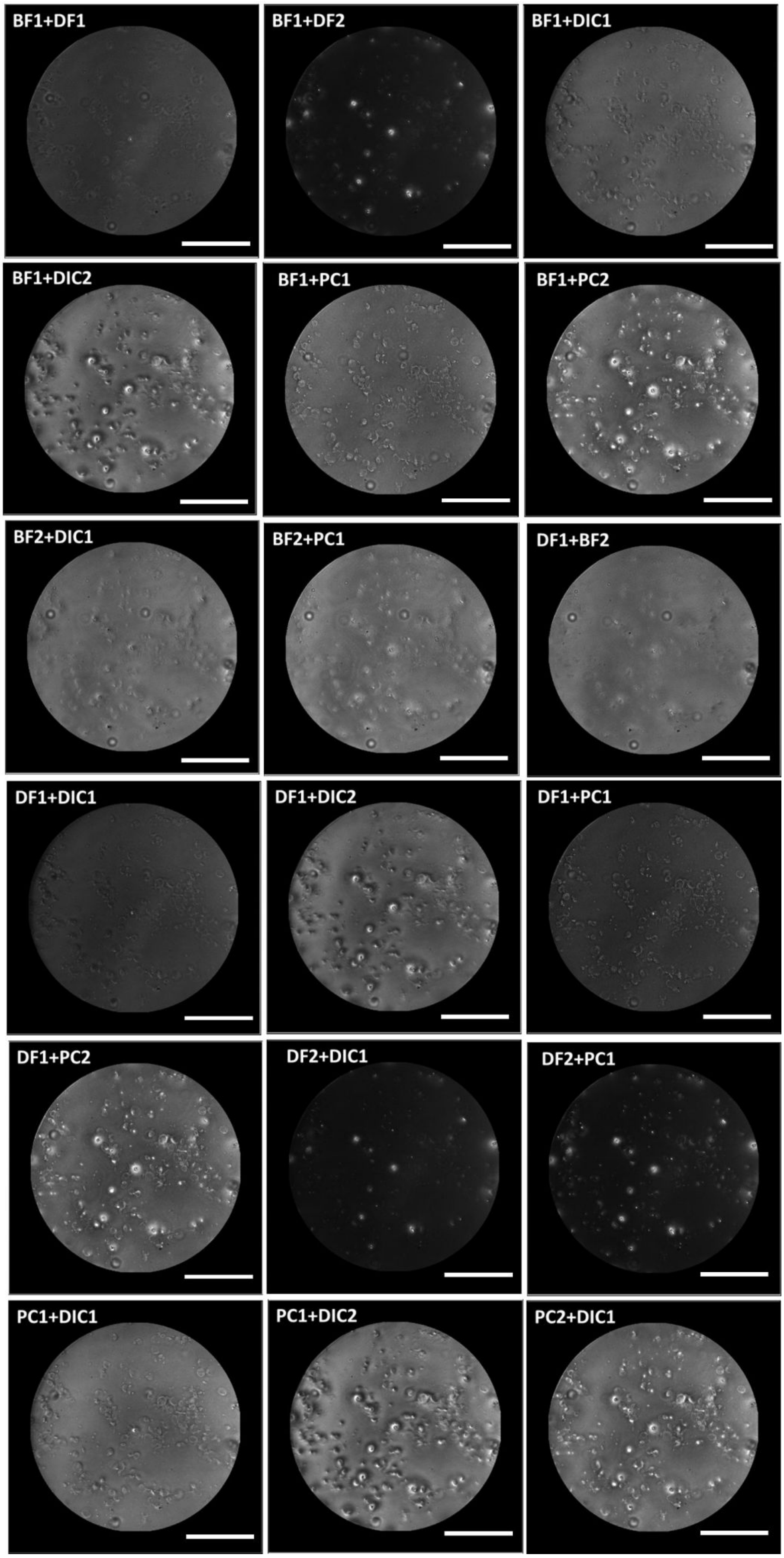
BF1+DF1
BF1+DF2
BF1+DIC1
BF1+DIC2
BF1+PC1
BF1+PC2
BF2+DIC1
BF2+PC1
DF1+BF2
DF1+DIC1
DF1+DIC2
DF1+PC1
DF1+PC2
DF2+DIC1
DF2+PC1
PC1+DIC1
PC1+DIC2
PC2+DIC1

Figure S2. The images combined two basic contrast techniques, including DIC+PC, DIC+BF, DIC+DF, PC+BF, PC+DF, and BF+DF. For each contrast technique, we selected two images, including DIC1, DIC2, PC1, PC2, DF1, DF2, BF1, and BF2. Afterward, we combined two images from two different contrast techniques to become BF1+DF1, BF1+DF2, BF1+DC1, BF1+DIC2, BF1+PC1, BF1+PC2, BF2+DDIC1, BF2+PC1, DF1+BF2, DF1+DIC1, DF1+DIC2, DF1+PC1, DF1+PC2, DF2+DIC1, DF2+PC1, PC1+DIC1, PC1+DIC2, and PC2+DIC1. The scale bar is 100 μm.
